# Work system barriers to & resilience strategies for COVID-19 PPE use in the emergency department: A qualitative interview study

**DOI:** 10.1017/ash.2022.71

**Published:** 2022-05-16

**Authors:** Oluseyi Daodu, Ayse Gurses, Patience Osei, Esosa Nosakhare, Shawna Perry, Marium Sultan, Nivedha Prabhu, Susan Peterson, Emma MacIntyre, Khue Vo, Lauren Yuan, Lauren Benishek, Jessica Li

## Abstract

**Background:** Emergency departments (EDs) are complex, sociotechnical, high-paced, safety-critical work systems that have been disproportionately affected by the COVID-19 pandemic. Despite training, consistent compliance with recommended PPE use during COVID-19 pandemic has been challenging. Healthcare workers (HCWs) have had adapt to overcome these challenges to ensure their own safety and patient safety. We sought to identify barriers in the work system that impede the recommended COVID-19 PPE use in EDs. **Methods:** We conducted semistructured, in-depth interviews over ZoomTM from August 2020–May 2021 with 45 HCWs from the ED (ie, physicians, nurses, ancillary support staff, etc) affiliated with a large, tertiary-care, academic medical center. These audio-recorded interviews were transcribed and analyzed using a hybrid (inductive and deductive) qualitative coding approach in NVivo software. The deductive portion was guided by the SEIPS work system model, a well-known human-factors conceptual framework. **Results:** We identified multiple work-system factors in the ED that impede compliance with the recommended COVID-19 PPE use. In addition, ED HCWs have reported making a variety of adaptations or developing strategies to overcome these barriers. Some of these adaptations were made to the PPE physically (eg, trimming portions of PPE), and others were related to the tasks and/or processes associated with PPE, such as filming their own training video demonstrating PPE donning and doffing techniques, and environment services staff checking a patient’s status with nurses prior to entering the patient’s room when there was no COVID-19 signage on the door. **Conclusions:** Consistent compliance with COVID-19 PPE use in ED clinical practice is challenging and can be negatively affected by a variety of work system factors. Resilience strategies developed by HCWs can provide critical information with regards to HCW needs and potential directions for innovation. Future efforts should focus on not only changing individual HCW behavior through training but also on improving the PPE and ED work system design.

**Funding:** US CDC

**Disclosures:** The authors gratefully acknowledge the US CDC for funding this work. This material is based upon work supported by the Naval Sea Systems Command (under contract no. N00024-13-D-6400, task order NH076). Any opinions, findings, and conclusions or recommendations expressed in this material are those of the authors and do not necessarily reflect the views of the Naval Sea Systems Command (NAVSEA) or the US CDC.

